# Is CD47 an innate immune checkpoint for tumor evasion?

**DOI:** 10.1186/s13045-016-0381-z

**Published:** 2017-01-11

**Authors:** Xiaojuan Liu, Hyunwoo Kwon, Zihai Li, Yang-xin Fu

**Affiliations:** 1 Key Laboratory of Infection and Immunity of CAS, Institute of Biophysics, Chinese Academy of Sciences, Beijing, China; 2 Department of Pathology, UT Southwestern Medical Center, Dallas, Texas USA; 3 Department of Microbiology and Immunology, Hollings Cancer Center, Medical University of South Carolina, Charleston, SC USA; 4 First Affiliated Hospital, Zhengzhou University School of Medicine, Zhengzhou, China

**Keywords:** CD47, SIRPα, “Don’t eat me” signal, Cancer immunotherapy, Chemotherapy, Clinical trial, Dendritic cell, Macrophage

## Abstract

Cluster of differentiation 47 (CD47) (also known as integrin-associated protein) is a ubiquitously expressed glycoprotein of the immunoglobulin superfamily that plays a critical role in self-recognition. Various solid and hematologic cancers exploit CD47 expression in order to evade immunological eradication, and its overexpression is clinically correlated with poor prognoses. One essential mechanism behind CD47-mediated immune evasion is that it can interact with signal regulatory protein-alpha (SIRPα) expressed on myeloid cells, causing phosphorylation of the SIRPα cytoplasmic immunoreceptor tyrosine-based inhibition motifs and recruitment of Src homology 2 domain-containing tyrosine phosphatases to ultimately result in delivering an anti-phagocytic—“don’t eat me”—signal. Given its essential role as a negative checkpoint for innate immunity and subsequent adaptive immunity, CD47-SIRPα axis has been explored as a new target for cancer immunotherapy and its disruption has demonstrated great therapeutic promise. Indeed, CD47 blocking antibodies have been found to decrease primary tumor size and/or metastasis in various pre-clinical models. In this review, we highlight the various functions of CD47, discuss anti-tumor responses generated by both the innate and adaptive immune systems as a consequence of administering anti-CD47 blocking antibody, and finally elaborate on the clinical potential of CD47 blockade. We argue that CD47 is a checkpoint molecule for both innate and adaptive immunity for tumor evasion and is thus a promising target for cancer immunotherapy.

## Background

Cluster of differentiation 47 (CD47), also known as integrin-associated protein (IAP), is a ~50 kDa heavily glycosylated, ubiquitously expressed membrane protein of the immunoglobulin superfamily with a single IgV-like domain at its N-terminus, a highly hydrophobic stretch with five membrane-spanning segments and an alternatively spliced cytoplasmic C-terminus [1]. Each of the four alternatively spliced cytoplasmic tails exists in vivo at different frequencies (i.e., form 2 is the most abundant), but all lack a substantial signaling domain [2]. While CD47 was first identified as a membrane protein involved in β3 integrin-mediated signaling on leukocytes [3], it is now known to also interact with thrombospondin-1, signal regulatory protein-alpha (SIRPα), and others to regulate various cellular functions including cell migration, axon extension, cytokine production, and T cell activation [4–8]. However, recent studies have focused most on CD47-SIRPα axis for its inhibitory role in phagocytosis [9]. SIRPα, also known as Src homology 2 domain-containing protein tyrosine phosphatase substrate 1/brain Ig-like molecule with tyrosine-based activation motif/cluster of differentiation antigen-like family member A (SHPS-1/BIT/CD172a), is another membrane protein of the immunoglobulin superfamily that is particularly abundant in the myeloid-lineage hematopoietic cells such as macrophages and dendritic cells [10, 11]. The ligation of SIRPα on phagocytes by CD47 expressed on a neighboring cell results in phosphorylation of SIRPα cytoplasmic immunoreceptor tyrosine-based inhibition (ITIM) motifs, leading to the recruitment of SHP-1 and SHP-2 phosphatases. One resulting downstream effect is the prevention of myosin-IIA accumulation at the phagocytic synapse and consequently inhibition of phagocytosis [12–14]. Thus, CD47-SIRPα interaction functions as a negative immune checkpoint to send a “don’t eat me” signal to ensure that healthy autologous cells are not inappropriately phagocytosed. Consistent with this notion, CD47^−/−^ cells are cleared rapidly when they are adoptively transferred to the congeneic wild-type mice [15]. However, it was recently shown that CD47-SIRPα axis, while crucial, represents just one mechanism that controls phagocytic behavior [16]. Indeed, CD47^−/−^ mice do not manifest significant self-destruction phenotype unless they are in inflammatory conditions. Inflammatory cytokines stimulate protein kinase C-spleen tyrosine kinase (PKC-Syk) signaling pathway (which IL-10 negatively regulates), which then activates macrophage to target self cells [16]. Combined, these findings suggest a potential mechanism for anemia of chronic disease and that rhesus (Rh)-null individuals, who have <25% of normal CD47 levels, may be particularly vulnerable to anemia under inflammatory conditions and infections [17].

Research has demonstrated overexpression of CD47 in nearly all types of tumors, some of which include acute myeloid leukemia, non-Hodgkin’s lymphoma, bladder cancer, and breast cancer [18–25]. While CD47 is implicated in the regulation of cancer cell invasion and metastasis [18, 26], its most well-studied and important function related to tumor development is prevention of phagocytosis via ligating with SIRPα on the surrounding phagocytes [18, 27, 28]. Also, CD47 expression on cancer stem cells (CSCs) implies its role in cancer recurrence. Particularly, a study has shown that CSCs have increased CD47 expression to protect themselves from immune-mediated elimination during conventional anti-tumor therapies [29]. This increases the chance of CSC survival, which in turn could repopulate a new tumor mass and cause a tumor relapse.

### CD47 blockade for direct cancer cell killing

Given the important inhibitory function of CD47 in phagocytosis of tumor cells, it has been extensively investigated as a potential target for tumor therapy. In various xenograft tumor models using *NOD-scid-IL2Rgamma* ^*null*^ (NSG) mice, use of human CD47-blocking monoclonal antibodies has demonstrated superb efficacy against human acute lymphocytic leukemia, acute myeloid leukemia, leiomyosarcoma, and solid tumors [18, 20, 27, 28, 30, 31]. Most work initially concluded that the therapeutic effects of anti-human CD47 were dependent on the *direct* killing of the tumor by phagocytes. However, it is important to note that xenograft models might have some unique features that favor innate immune-mediated tumor killings. First, human CD47 binds well to SIRPα of NSG mice, but not of other strains [32, 33]. This unique feature could put human tumor cells under CD47-SIRPα control more so in NSG mice than in other strains of mouse, making them more susceptible to signaling blockade. Thus, use of human SIRPα-transgenic recombination-activating gene (Rag)2^−/−^ IL2Rgamma^−/−^ mice may be necessary to accurately test such antibody’s therapeutic benefit [34]. Second, in xenograft models, only human tumor cells express human CD47. Hence, human CD47-blocking monoclonal antibodies can efficiently target human tumors without being “absorbed” by other normal cells (such as red blood cells) expressing mouse CD47. Third, xenograft tissue could come under strong innate immune attack. For example, lacking the mouse MHC class I “self” marker, xenograft human tumor cells might be attacked by natural killer (NK) cells if human leukocyte antigen (HLA) fails to mediate inhibitory signaling. Consistent with this notion, in syngeneic immunodeficient mouse models such as athymic nude mice or *Rag*-deficient mice, mouse anti-CD47 blockade resulted in less impressive efficacy after treatment [35]. Fourth, lymphocyte-deficient mice typically demonstrate stronger innate immune responses [36]. All reasons listed above suggest that contribution of direct killing by phagocytes to the therapeutic impact of CD47 blockade may be significantly different in an immunocompetent organism.

### Role of CD8^+^ T cells upon CD47 blockade

Indeed, adaptive immune response, particularly that mediated by T cells, plays an important role in mouse anti-CD47 blockade-induced tumor control. In syngeneic immunocompetent mouse models, mouse anti-CD47 blockade shows impressive anti-tumor effect especially upon intratumoral delivery [35, 37]. Depletion of CD8^+^ T cells—but not CD4^+^ T cells—diminishes the therapeutic effect of anti-mouse CD47 antibody. Furthermore, after anti-mouse CD47 treatment, significantly more interferon (IFN)-γ spot-forming antigen-specific CD8^+^ T cells are present in the tumor, and T cell-mediated memory response is formed to protect mice from tumor re-challenge. All of these experimental results demonstrate that T cells are essential for anti-mouse CD47-mediated tumor regression. Thus, CD47 is a checkpoint molecule for both innate and adaptive immunity for tumor evasion.

### Role of dendritic cells upon CD47 blockade

Since macrophages have been shown to play an important role in tumor cell phagocytosis in the xenograft model, they were assumed to be the major antigen-presenting cells for cytotoxic T lymphocyte (CTL) induction. Supporting this, enhancement of cross-priming by macrophages was observed in response to anti-human CD47 treatment [38]. However, using the syngeneic mouse model, we have recently shown that dendritic cells—not macrophages—appeared to play a more important role for CTL cross-priming and anti-tumor therapy based on the following observations [35]. First, in the presence of anti-mouse CD47 antibody, bone marrow-derived dendritic cells (BMDCs) were able to cross-prime CD8^+^ T cells to a greater extent than bone marrow-derived macrophages (BMDMs) in general. Second, ex vivo isolated dendritic cells (DCs) were more potent for cross-priming of CTL than macrophages after anti-mouse CD47 treatment. Third, the therapeutic effect of anti-mouse CD47 antibody was severely impaired following DC depletion but not macrophage depletion. Apparent contradiction between the two studies likely resulted from differences in experimental approaches. Indeed, when BMDCs were cultured without serum (similar to in vitro phagocytosis/priming assays in [38]), they demonstrated increased apoptosis (as measured by increased annexin V stain) which would likely impact their functional capacity. In contrast, macrophages demonstrated very minimal change in annexin V stain in the presence/absence of the serum [35].

Also, it seems that although macrophages can phagocytose more tumor cells, DCs are more potent than macrophages in antigen presentation [39]. Macrophages are good at scavenging and destroying phagocytosed tumor cells, but at the same time, tumor antigens and danger signals are overly degraded [39]. In contrast, DCs have developed means to preserve useful information from the ingested tumor cells that serve to initiate adaptive immune responses [39].

**Fig. 1:**
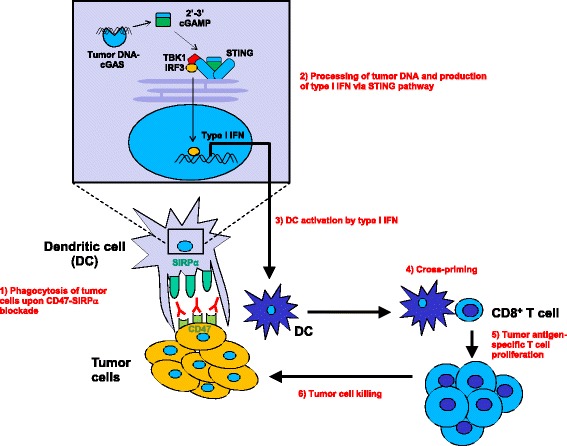
Working model of CD47 blockade for enhancing antigen cross-presentation by dendritic cells and increased T cell priming. Upon CD47-SIRPa blockade, tumor cells are phagocytosed and their DNA can gain access to the cytosol of intratumoral dendritic cells. Recognition of cytosolic DNA by cyclic GMP-AMP (cGAMP) synthase (cGAS) and generation of cGAMP lead to the activation of STING, resulting in the production of type I IFN. DCs are activated by type I IFN to cross-present tumor antigens to CD8^+^ T cells, which then proliferate and kill tumor cells

How anti-CD47 blockade boosts DC-mediated antigen cross-presentation and CTL induction is an intriguing question that we have started to answer. We found that after anti-mouse CD47 treatment, DCs—but not macrophages—express more *Ifna* mRNA [35]. Blocking type I IFN signaling by intratumoral injection of interferon alpha/beta receptor (IFNAR)-blocking antibody impaired the therapeutic effect of anti-mouse CD47, suggesting an important role of type I IFN signaling on DC activation. Supporting this, conditional deletion of *Ifnar* 1 in CD11c^+^ cells markedly reduced the therapeutic effect of CD47 blockade on tumor growth. These data also confirm the essential role of DCs as antigen-presenting cells (APCs) in vivo for CTL induction. Interestingly, our data further demonstrated that cytosolic DNA sensor stimulator of interferon genes (STING)—but not classical Toll-like receptor (TLR)-myeloid differentiation primary response gene 88 (MyD88) pathway—is required for type I IFN production and the therapeutic effect of anti-CD47. This raises a fascinating scenario that upon anti-CD47 treatment, DNA is released from tumor cells and taken up by DCs, resulting in the activation of STING and the production of type I IFN, which activates DCs for antigen cross-presentation (Fig. 1). The detailed mechanisms remain to be investigated in the future.

### Targeting CD47-SIRPα signaling axis for therapy

As of November 13, 2016, there are eight phase I clinical trials that are investigating the effect of blocking CD47-SIRPα signaling axis in various cancer patients (summarized in Table 1). Among the six, NCT02216409, led by Forty Seven, Inc., is the first in-human trial and the *only one* yet whose data have been presented [40]. Briefly, in this study, humanized monoclonal anti-CD47 antibody (“Hu5F9-G4”) [41] was administered to patients with diverse solid tumors who are no longer candidates for conventional therapies. As a phase I clinical trial, it sought to determine the appropriate dosage of Hu5F9-G4 and to perform the initial pharmacodynamic and -kinetic studies. Patients tolerated priming (starting) dose of 0.1, 0.3, and 1 mg/kg well, while those receiving 3 mg/kg experienced a dose-limiting toxicity (abdominal pain, RBC hemagglutination, and headache). Hence, 1 mg/kg was decided as the priming dose, and currently, work is being done to determine the optimal maintenance dose. Hu5F9-G4-related adverse events, majority of which were reversible, included anemia, hyperbilirubinemia, headache, hemagglutination, nausea, and retinal toxicity. It would be interesting to see in the future how other two therapeutic agents compare to Hu5F9-G4 in terms of their safety profiles.

**Table 1:** List of CD47-SIRPα axis-blocking therapeutic agents that are currently being tested in phase I interventional clinical trials

Therapeutic agent(s)	Details of the therapeutic agent	Clinical trial identifiers	Study start date	Disease in recruited patients
Hu5F9-G4 (NCT02953782 and NCT02953509 use cetuximab and rituximab, respectively, in combination)	• Humanized anti-human CD47 monoclonal antibody • Generated by grafting complementarity determining region (CDR) onto a human IgG4	1. NCT02216409 2. NCT02678338 3. NCT02953782 4. NCT02953509	1. August 2014 2. November 2015 3. November 2016 4. November 2016	1. Solid malignancy 2. Acute myeloid leukemia 3. Solid malignancy and advanced colorectal cancer 4. Relapsed/refractory B cell non-Hodgkin’s lymphoma
TTI-621	• A soluble recombinant SIRPα-Fc fusion protein • Generated by combining the sequences encoding the N-terminal portion of human SIRPα with the Fc region of human IgG1	1. NCT02890368 2. NCT02663518	1. October 2016 2. January 2016	1. Solid malignancy and mycosis fungoides 2. Hematologic malignancy
CC-90002	• Monoclonal anti-human CD47 antibody	1. NCT02367196 2. NCT02641002	1. March 2015 2. March 2016	1. Solid and hematologic malignancies 2. Acute myeloid leukemia and myelodysplastic syndrome

It is still unclear, however, if administration of Hu5F9-G4 alone will result in therapeutic benefits that are expected based on the promising results of many pre-clinical studies. Indeed, effective clinical responses are generally rare and statistically inconclusive in phase I trials, mainly due to small numbers of patients and inability to optimally administer the therapeutic agent (i.e., the dosage). Phase II and III trials will be critical for evaluating the ability to either delay disease progression or perhaps even cause its remission.

Given that blockade of CD47-SIRPα signaling axis has (and continues to) demonstrate success in more pre-clinical tumor models, more entries into clinical trials involving the CD47-SIRPα axis are anticipated. Below, we offer some suggestions and important considerations to potentially improve the specificity and efficacy of therapy.

### Chemotherapy influences anti-mouse CD47 effects

Many patients might have previously received or continue to receive chemotherapy during anti-CD47 treatment. Since chemotherapy can suppress the immune system by killing recently activated immune cells [42, 43], it is possible that chemotherapy may blunt the therapeutic effects of CD47 blockade. However, on the other hand, chemotherapy may increase the release of tumor antigen and DNA from dying tumor cells, which may synergize with CD47 blockade. These possibilities have been experimentally evaluated [35]. It was found that chemotherapy administered after anti-CD47 therapy has a detrimental effect on the development of beneficial anti-tumor memory immune responses. In contrast, chemotherapy administered before anti-CD47 therapy not only synergized with anti-CD47 for tumor control but also preserved the host memory response against relapsing tumors. Several possibilities exist for the synergistic effect of chemotherapy and anti-CD47 treatment. First, chemotherapy may induce the release of tumor DNA from dying tumor cells, which could augment STING-mediated cytosolic DNA sensing. Second, chemotherapy may sensitize tumor cells by the upregulation of “eat me” signals, such as surface calreticulin, which could synergistically amplify the CTL induction in combination with “don’t eat me” blockade. Third, it is also possible that the chemotherapy preconditions the tumor microenvironment with more infiltrating inflammatory cells, allowing anti-CD47 blockade to work. Therefore, proper combination therapy of chemotherapeutic drugs and anti-CD47 antibody may depend on the type, timing, dose of these agents, and tumor types. Further studies are needed to uncover the underlying synergistic mechanisms for a rational combinational design.

### Intratumoral CD47-SIRPα blockade

Given the ubiquitous expression of CD47 on normal cells, tumor-specific delivery of CD47 blockade would generate better anti-tumor effect with fewer side effects than systemic administration. Indeed, the possibility of attack against healthy self cells warrants a concern. For example, patients, especially those under chronic inflammatory conditions or infection, may become severely anemic upon CD47 blockade [16]. Thus, how to block CD47-SIRPα inside the tumor tissues specifically becomes the challenge. Tumor-targeting antibodies may be conjugated with anti-CD47 or SIRPα-Ig to increase specificity [44]. In the selection of a conjugation partner, two kinds of partners can be exploited. One is pro-phagocytic Fc receptor (FcR)-activating antibodies, such as the anti-CD20 antibody, since CD47-SIRPα interruption can synergize with antibody-dependent cellular phagocytosis [20, 44]. The other partner can be adaptive check point blockade antibodies including anti-programmed death ligand 1 (PDL1) for unleashing both an innate and adaptive anti-tumor response [45]. While cytotoxic T lymphocyte-associated protein 4 (CTLA4) or programmed cell death protein 1 (PD1) blockade monotherapy has gained enormous attention for its potential to result in a durable clinical response and prolonged overall survival with tolerable toxicity compared to standard chemotherapy, not all patients respond [46]. Discovery that nivolumab and ipilimumab dual therapy is more efficacious than ipilimumab monotherapy in patients with untreated metastatic melanoma highlights the importance of combination therapy and search for other molecular targets [47]. It is possible that combination therapy of anti-CD47 antibody, which increases the tumor cell phagocytosis and priming of anti-tumor CD8^+^ T cell responses, and anti-CTLA4/PD1, which reinvigorates exhausted T cells, may give greater synergism by improving different steps to generating effective anti-tumor immunity. Such idea that tumor-targeted delivery of the CD47 checkpoint antagonist can work as a potential booster to synergize with other tumor-targeting antibodies for better cancer immunotherapy is being actively investigated, as reflected by phase I clinical trials testing its combination therapy with cetuximab or rituximab (Table 1).

## Conclusions

Many solid and hematologic malignancies express CD47 on their cell surface to display an anti-phagocytic signal to SIRPα-expressing myeloid cells and evade destruction by innate and adaptive immune system. Administration of anti-CD47 blocking antibodies has been enormously successful in various pre-clinical models, mechanism of which likely involves both phagocyte-mediated direct killing and their cross-priming of cytotoxic T cells. Our recent work has illustrated a critical role for dendritic cells and the STING pathway, as well as CD8^+^ T cells, to achieving therapeutic effect of CD47 blockade. Currently, there are eight clinical trials in progress related to CD47-SIRPα blockade and more entries are anticipated. In the future, a combinational design including anti-CD47 antibody with appropriate chemotherapy and immune-modulating agents such as anti-tumor antibodies, type I IFN, STING agonists, immune checkpoint modulators, and others should be intensely investigated for achieving synergistic and tumor-specific effect for clinical application.

## Abbreviations

APCs: Antigen-presenting cells
BMDC: Bone marrow-derived dendritic cells
CD47/IAP: Cluster of differentiation 47/integrin-associated protein
cGAMP: Cyclic GMP-AMP
cGAS: cGAMP synthase
CSC: Cancer stem cell
CTL: Cytotoxic T lymphocyte
CTLA4: Cytotoxic T lymphocyte-associated protein 4
DCs: Dendritic cells
DNA: Deoxyribonucleic acid
FcR: Fc receptor
GMP-AMP: Guanosine-adenosine monophosphate
HLA: Human leukocyte antigen
IFN: Interferon
IFNAR: Interferon alpha/beta receptor
Ig: Immunoglobulin
IL10: Interleukin 10
ITIM motifs: Immunoreceptor tyrosine-based inhibition motifs
MHC: Major histocompatibility complex
mRNA: Messenger ribonucleic acid
MyD88: Myeloid differentiation primary response gene 88
NK: Natural killer
NSG: *NOD-scid-IL2Rgamma* ^*null*^
PD1: Programmed cell death protein 1
PDL1: Programmed death ligand 1
PKC: Protein kinase C
RAG: Recombination-activating gene
Rh: Rhesus
SIRPα/SHPS1/BIT/CD172a: Signal regulatory protein-alpha/Src homology 2 domain-containing protein tyrosine phosphatase substrate 1/brain Ig-like molecule with tyrosine-based activation motif/cluster of differentiation antigen-like family member A
STING: Stimulator of interferon genes
Syk: Spleen tyrosine kinase
TLR: Toll-like receptor
